# Salmonella typhi Meningitis in an Immunocompetent Asian Adult

**DOI:** 10.7759/cureus.6112

**Published:** 2019-11-10

**Authors:** Rashid Nadeem, Naheed Elahi, Ashraf Elhoufi, Ahmed Elsousi, Monazza Chaudhry

**Affiliations:** 1 Intensive Care Medicine, Dubai Hospital, Dubai, ARE; 2 Internal Medicine, Hennepin Healthcare, Minneapolis, USA

**Keywords:** meningitis, salmonella, typhi, bacteremia, morbidity, mortality, external ventricular drain

## Abstract

Enteric salmonella infections are common in the United States though nonenteric salmonella infections in immunocompetent adults are exceedingly rare in the United States, and meningitis is one of the least common extra-intestinal sites. In addition, it is very unusual for a patient with bacterial meningitis to present with meningitis signs and symptoms of >72 h duration like aseptic meningitis.

A 25-year-old Indian male, without any past medical history brought by friends to the ER had fever and irritability for a week. He became increasingly somnolent and confused three days back. Physical exam reveals signs of meningitis and laboratory showed cerebrospinal fluid (CSF) studies suggestive of bacterial meningitis. Culture of CSF grew Salmonella typhi and later on blood culture also grew S. typhi. The patient became worse with cerebral edema and hydrocephalous suspected by change in neurological status and confirmed by ultrasound of eye ball examining optic nerve sheath diameter and CT scan of brain. The patient required neurosurgical intervention and hence external ventricular drain was placed. The patient was also placed on mechanical ventilation. Subsequently he developed ventilator associated pneumonia (VAP) with carbapenem-resistant Pseudomonas aeruginosa. The patient underwent tracheostomy and successfully completed treatment of VAP and was transferred to his home country after four months. Thus we describe a rare case of salmonella meningitis in an immunocompetent adult.

## Introduction

Salmonella infection is a well-known cause for food-borne diarrhea worldwide [1]. Incidence of salmonella meningitis is rare, only 4-6 per 100,000/year especially in immunocompetent hosts [2]. It is frequently a medical emergency which may require neurosurgical intervention and may result in substantial morbidity and mortality despite optimal therapy [3]. It mainly affects children under five, especially infants [4]. In adult population salmonella meningitis is predominantly reported in immunocompromised [5]. The incidence of salmonella meningitis in immunocompetent adults in the Middle East is unknown. Medical literature reveals only case reports [6]. We describe such a case of salmonella meningitis in an immunocompetent adult male.

## Case presentation

A 25-year-old Indian male, without any past medical history brought by his friends to the ER had fever and irritability for a week. He became increasingly somnolent and confused three days back. At the time of presentation, he also had neck pain, stiffness, generalized weakness, and headache. Family denied recent travel, sick contacts, high-risk sexual exposures, and any recent gastrointestinal symptoms.

Physical exam reveals dry mucous, membranes, a stiff neck with decreased range of motion, and a positive Kernig’s sign but no obvious Brudzinski’s sign; pupils were 4 mm bilaterally equal and reactive to light. He was tachycardic and hyperdynamic but regular abdominal and extremity examinations were unremarkable; on neurological examination, he was lethargic but responded appropriately to commands, was oriented, and moving all extremities; he had unsteady gait. He had no rashes, petechiae, or purpuric lesions.

Laboratory data showed WBC 3700 /micro L; procalcitonin was 28 ng/mL, CRP was 220 mg/L; cerebrospinal fluid (CSF) studies showed protein 458 mg/dL, glucose <2 mg/dL, chloride 113 mg/dL, lactic acid 17.1 with CSF WBC count; 107/cmm, RBC 2750/cmm. CSF Gram stain showed Gram negative bacilli and culture showed *Salmonella typhi*; later blood culture also grew *S. typhi*.

**Figure 1:**
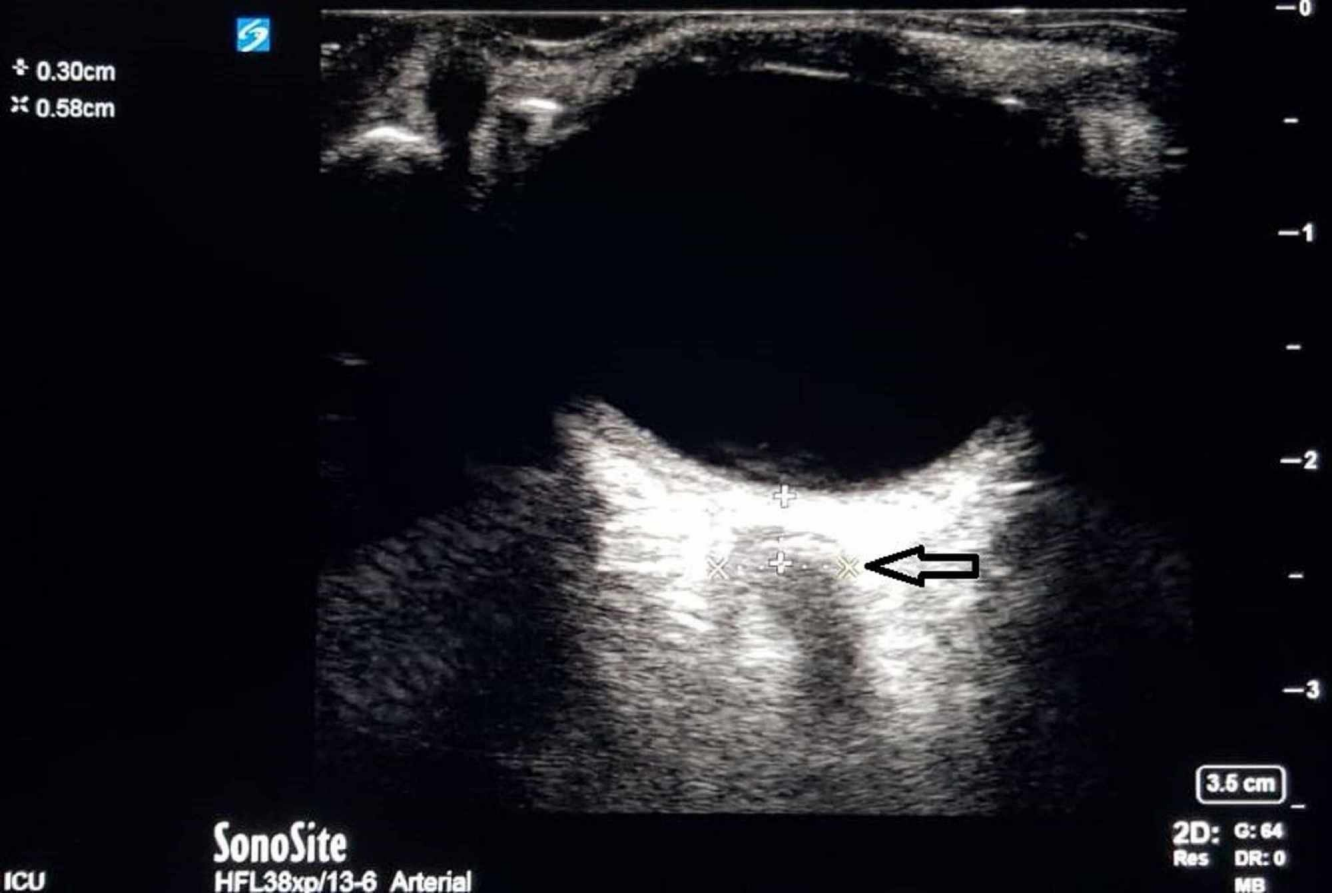
Ultrasound eye performed after 30 h of admission showing optic nerve sheath diameter (distance between two XX) as shown by black arrow.

**Figure 2:**
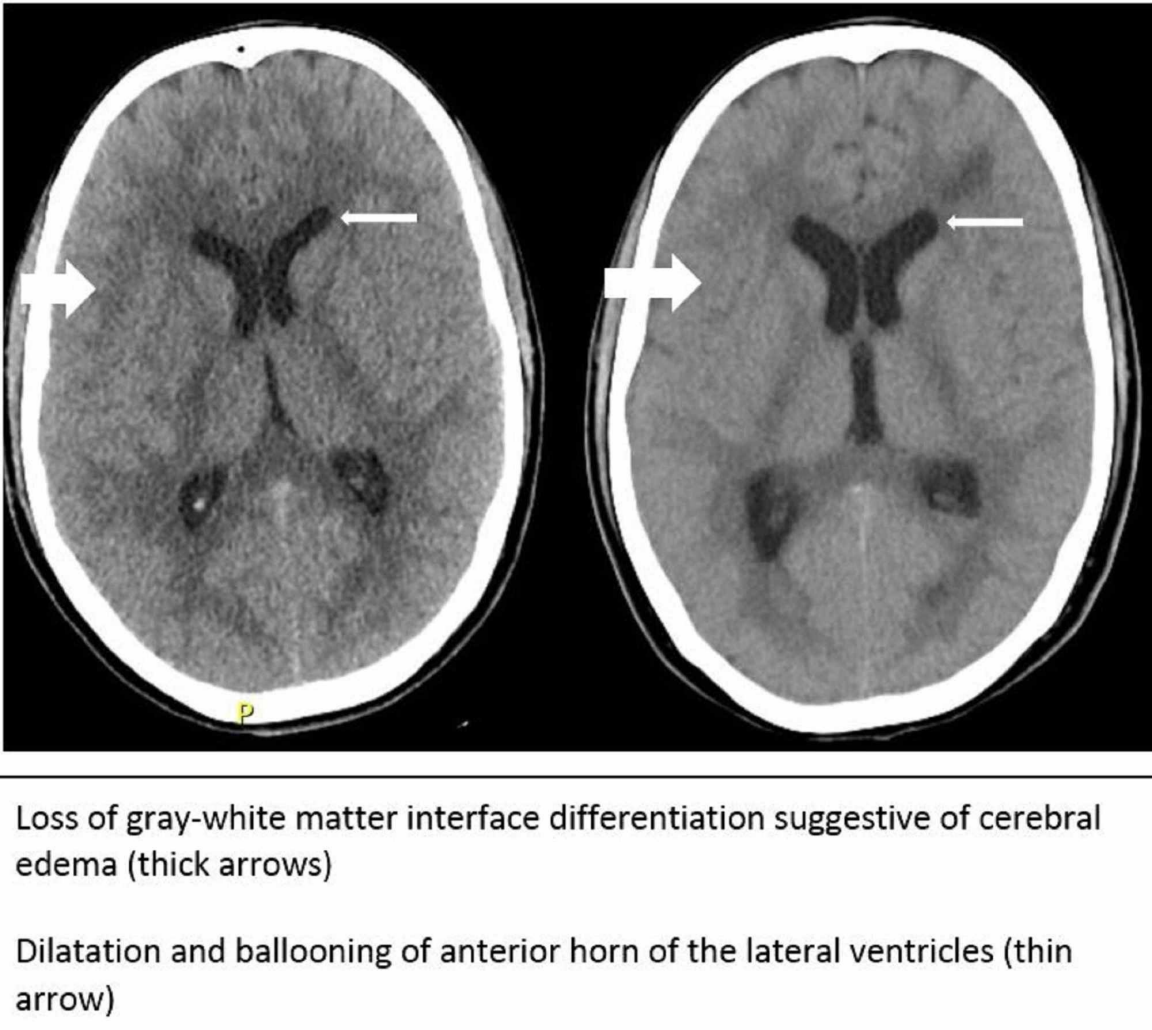
Development of cerebral edema and hydrocephalous; (left CT scan) before and (right CT scan) evaluated by neurosurgeon and radiologist.

Radiological imaging of brain showed multiple hypo densities seen in the deep white matter of frontal lobe, occipital lobe, and left parietal lobe. He was diagnosed as a case of meningoencephalitis, and started on antibiotics. The patient’s Glasgow coma scale (GCS) worsened from 8/15 then to 6/15 and pupils became unequal and nonreactive. An ocular ultrasound was done to measure optic disc which was abnormal (5.8 mm) (Figure 1), so a repeat CT brain was done which showed subtle brain parenchymal hypo density in both occipital lobes, suggestive of evolving ischemic changes, and persisting cerebral edema, diffuse hydrocephalous (Figure 2) and patchy deep white matter hypo densities in frontal and parietal lobes; therefore an external ventricular drain was placed. The patient was placed on mechanical ventilation as well. Subsequently he developed ventilator associated pneumonia (VAP) with carbapenem-resistant *Pseudomonas aeruginosa*. Later on the patient underwent tracheostomy and successfully completed treatment of VAP and transferred to his home country after about four months.

## Discussion

Salmonella infections can be enteric or nonenteric focal (abscesses, osteomyelitis, mycotic aneurysms, septic arthritis, pneumonia, endocarditis, and meningitis meningitis) or systemic (bacteremia). Our patient had both meningitis and bacteremia. These focal infections are most commonly seen in immunocompromised patients, i.e. HIV positive patients [5] or patients having immune suppressing medication like steroids [7] or tumor necrosis factor antagonist [8] or a previously presumed immunocompetent patient later found as immunosuppression, i.e. AIDS [9]. Rarely it infects immunocompetent patient who has structural abnormalities or chronic conditions such as malignancy, diabetes, or sickle cell disease [10]. Similar cases have been reported in the United States [11] and outside the United states (in Pakistan) as CSF culture proven meningitis or clinical meningitis with CSF pleiocytosis (CSF culture negative) with salmonella bacteremia [12] or in Sweden with CSF culture positive for *Salmonella virchow* [13].

Clinical presentation in reported cases and our case was similar. Majority have two out of three symptoms; fever, neck stiffness, and altered mental status [3]. All three symptoms are present only in two third of patients. Onset is gradual with prolonged course (one week of symptoms) more suggestive of picture like aseptic meningitis. Therefore, most have an extended period of symptoms before definitive intervention. This delay may lead to high morbidity and mortality rate (40%-70%) [6, 10].

Early detection of intracranial hypertension (ICH) can be helpful in these patients. Lumber puncture is the gold standard to diagnose ICH. Abnormalities in coagulation status, thrombocytopenia, and anticoagulation may not allow this invasive procedure. CT scan requires shifting of patient to radiology department. Therefore, noninvasive bedside ultrasonography has emerged as an attractive alternative to diagnose and monitor ICH. Ultrasonography of optic nerve sheath diameter (ONSD) shows a good level of diagnostic accuracy for detecting intracranial hypertension [14-15]. 

Management includes empiric treatment of bacterial meningitis; third-generation cephalosporin and vancomycin. Ampicillin is added for specific subgroups at risk for Listeria meningitis [16], not common in the Middle East. Our patient was treated with ceftriaxone and levofloxacin. Duration of antimicrobial therapy is an important factor in prevention of recurrence as there are reported incidences of recurrence of salmonella meningitis in patients whose antibiotics were stopped after two to three weeks [17].

Salmonella meningitis is frequently complicated with intracranial abscesses [15], cerebral edema [16], and hydrocephalous as in our patient. Mahapatra et al. described a case series of six pediatric patients with sudural empyema requiring drainage [18]. When patients are presenting with cerebral abscess which cannot be drained, it is recommended to treat with broad spectrum antibiotics for at least four to five weeks duration or until all the abscesses in the brain have resolved on radiological imaging [17, 19].

## Conclusions

We described this case of *S. typhi* meningitis in immunocompetent adults. The patient presented after several days of symptoms which highlights the need for a good clinical perspective; if a patient appears to have clinical picture of bacterial meningitis suggested by high fever, neck stiffness, headache, and change in mental status -- optimal management should be instituted without delay and continued until objective data supporting or excluding the diagnosis becomes available. Clinical course of our patient also documents the associated morbidity requiring neurosurgical intervention, therefore, periodic neurological evaluation by serial exams, optic ultrasound (ONSD), and CT scans are warranted for any significant change in clinical condition.
